# Targeted Sequencing of *FKBP5* in Suicide Attempters with Bipolar Disorder

**DOI:** 10.1371/journal.pone.0169158

**Published:** 2016-12-28

**Authors:** Marie E. Breen, Sophia C. Gaynor, Eric T. Monson, Kelly de Klerk, Meredith G. Parsons, Terry A. Braun, Adam P. DeLuca, Peter P. Zandi, James B. Potash, Virginia L. Willour

**Affiliations:** 1 Department of Psychiatry, University of Iowa Carver College of Medicine, Iowa City, Iowa, United States of America; 2 Department of Ophthalmology and Visual Sciences, University of Iowa Carver College of Medicine, Iowa City, Iowa, United States of America; 3 Department of Biomedical Engineering, University of Iowa College of Engineering, Iowa City, Iowa, United States of America; 4 Department of Mental Health, Johns Hopkins Bloomberg School of Public Health, Baltimore, Maryland, United States of America; University of Michigan, UNITED STATES

## Abstract

FKBP5 is a critical component of the Hypothalamic-Pituitary-Adrenal (HPA) axis, a system which regulates our response to stress. It forms part of a complex of chaperones, which inhibits binding of cortisol and glucocorticoid receptor translocation to the nucleus. Variations in both the HPA axis and *FKBP5* have been associated with suicidal behavior. We developed a systematic, targeted sequencing approach to investigate coding and regulatory regions in or near *FKBP5* in 476 bipolar disorder suicide attempters and 473 bipolar disorder non-attempters. Following stringent quality control checks, we performed single-variant, gene-level and haplotype tests on the resulting 481 variants. Secondary analyses investigated whether sex-specific variations in *FKBP5* increased the risk of attempted suicide. One variant, rs141713011, showed an excess of minor alleles in suicide attempters that was statistically significant following correction for multiple testing (Odds Ratio = 6.65, P-value = 7.5 x 10^−4^, Permuted P-value = 0.038). However, this result could not be replicated in an independent cohort (Odds Ratio = 0.90, P-value = 0.78). Three female-specific and four male-specific variants of nominal significance were also identified (P-value < 0.05). The gene-level and haplotype association tests did not produce any significant results. This comprehensive study of common and rare variants in *FKBP5* focused on both regulatory and coding regions in relation to attempted suicide. One rare variant remained significant following correction for multiple testing but could not be replicated. Further investigation is required in larger sample sets to fully elucidate the association of this variant with suicidal behavior.

## Introduction

Every hour throughout the world, approximately ninety-one people die by suicide and reports suggest suicide attempts are over twenty times higher [1]. The complexity of attempted and completed suicide, collectively termed suicidal behavior, is due to the combination of biological, behavioral and environmental factors that can increase the risk of this phenotype [2]. One of the most common risk factors is the presence of a psychiatric disorder, predominantly mood disorders such as bipolar disorder, with almost 90% of suicide attempters suffering from a preceding psychiatric condition [3, 4].

One current hypothesis in the field of suicide genetics is that suicidal behavior is associated with an altered stress response. The Hypothalamic-Pituitary-Adrenal (HPA) axis is a tightly controlled collection of interacting proteins that help regulate our response to stress. Dysregulation of the HPA axis causes an abnormal stress response and has been linked to suicidal behavior [5–7]. One critical component of the HPA axis is the co-chaperone FK506 Binding Protein 5 (FKBP5; ENSG00000096060). FKBP5 binds to the glucocorticoid receptor (GR) as part of a complex of chaperones. This complex inhibits binding of cortisol and receptor translocation to the nucleus [8]. Due to its crucial role in the HPA axis, specific focus has been placed on the *FKBP5* gene and variations within it that might be associated with suicidal behavior. A family-based study found an association with *FKBP5* and bipolar disorder, and a secondary analysis linked this to a history of attempted suicide [9]. European [10, 11] and Japanese [12] sample cohorts with suicidal behavior (ranging from suicidal ideation to attempt and completed suicide) have also been found to show associations with *FKBP5*. Adding more complexity, it has been suggested that *FKBP5* variants may interact with childhood trauma to influence suicidal behavior [13, 14]. The presence of regulatory variation within *FKBP5* may be functionally relevant as gene and protein expression studies have found altered FKBP5 levels in suicide completers compared to controls [15]. In contrast, genome-wide studies have not shown any association between *FKBP5* and suicidal behavior [16–27].

The conflicting nature of these results suggests that a more focused interrogation of this gene including less commonly investigated regions and less common variants, may identify functionally relevant variation. Therefore, in this study, we have sequenced both coding regions and regulatory regions to identify genetic variation in *FKBP5* that may increase the risk for suicidal behavior in individuals with bipolar disorder. Following stringent quality control checks, we performed single-variant, gene-level and haplotype tests on the resulting 481 variants. One rare variant remained significant following correction for multiple testing but could not be replicated. Further investigation is required in larger sample sets to fully elucidate the association of this variant with suicidal behavior.

## Materials and Methods

### Ethics Statement

Samples were obtained from the National Institute of Mental Health (NIMH) Genetics Initiative, (https://www.nimhgenetics.org/; S1 File) [28] and distributed through Rutgers University’s RUCDR Infinite Biologics (http://rucdr.org/). The University of Iowa Institutional Review Board considers this project to be “not human subject research” because these repository samples were sent to the University of Iowa’s laboratory in a de-identified manner.

### Subjects

For this study, we utilized the same sample set as in our attempted suicide genome-wide association study (GWAS) [16]. All subjects included in the analysis were European-American and unrelated. Phenotype data was collected using the Diagnostic Interview for Genetic Studies (DIGS; versions 1.0–4.0 [29]) and diagnoses ascertained as bipolar disorder, type I (BPI) or Schizoaffective Disorder, Bipolar Type (SABP). There was no significant difference in average age between suicide attempters and non-attempters (S1 Table).

### Definition of Suicide Subjects

Case subjects were defined as suicide attempters if they answered yes to the DIGS question: “Have you ever tried to kill yourself?” and had definite or serious intent to complete suicide. This resulted in a total of 476 suicide attempters and 476 non-attempters for sequencing.

### Population Stratification

Principal Component Analysis (PCA) was performed using EIGENSTRAT in EIGENSOFT v3.0 [30] on our entire attempted suicide GWAS [16], and these results were included as covariates in the logistic regression analyses.

### Target Determination

The UCSC Genome browser databases (UCSC Genes [31], GenCODE Genes [32], Ensembl Genes [33], RefSeq Genes [34] and ENCODE databases [35]) were used to define coding and regulatory regions in or near *FKBP5* transcripts. Targets included: **(i)** all exons (±50 bases) from all available transcripts (generated from the previously discussed UCSC Genome browser databases), **(ii)** predicted DNase hypersensitivity sites with at least one overlapping transcription factor binding site within all *FKBP5* transcripts (±10 kb) using ENCODE and **(iii)** promoter regions (determined as 2 kb upstream of all the transcript start sites). Coding regions encompass any translated exons while regulatory regions include all other loci covered.

### SureSelect Technology

We used a customized SureSelect Target Enrichment System (Agilent Technologies, Santa Clara, CA, U.S.A.) to capture and sequence our chosen targets (as previously described in [36]). Briefly, 3 μg genomic DNA was sheared, prepared into a library using adaptors and hybridized to biotinylated RNA baits. Pre-designed DNA targets were collected using magnetic streptavidin beads and sequenced as 100 bp paired-end reads using the HiSeq2000 at the Iowa Institute of Human Genetics Genomics Division in pools of sixteen samples.

### Sample Processing Pipeline

All samples were processed as a group. Samples were aligned to the Human Feb. 2009 Assembly (GRCh37/hg19) using the Burrows-Wheeler Aligner (BWA) tool v0.6.2 [37] and a 30 base seed length. Using SamTools v0.1.18 [38], aligned files were converted to binary (BAM) files and then sorted and indexed. Unpaired, improperly paired and unmapped reads were excluded using BamTools v2.2.3 [39] and duplicate reads removed using Picard v1.88 (http://picard.sourceforge.net). Further BamTool filtering included a mapping quality score > 20. Genome analysis toolkit (GATK) v3.1.1 [40] best practices were used to perform such tasks as realignment around insertion/deletions (indels), base score recalibration, variant calling using Haplotypecaller, and initial quality checks (such as strand bias and depth ratio). Any single nucleotide polymorphism (SNP) or indel which failed these checks was excluded. Variant calls were labeled as missing if they had a call depth < 10, or a genotyping quality Phred score < 20. Variants were excluded from the final dataset if they had missing calls in > 10% of subjects or a Hardy-Weinberg P-value < 1 x 10^−6^. Following a comparison to our previous GWAS genotypes, three non-attempters were excluded before analysis due to mismatches, resulting in a final total of 473 non-attempters for analysis. All variants were annotated using Ensembl v75 data-tracks via ANNOVAR (vMarch 22, 2015) [41].

### Statistical Analysis

On a single-variant level, we tested 481 *FKBP5* variants for an association with attempted suicide using a logistic regression test with Firth’s penalized maximum likelihood method via the logistf R package v1.21 [42].

On the gene level, we assessed the association of variation across the *FKBP5* locus with suicidal behavior, with two minor allele frequency (MAF) thresholds (≤ 1% and ≤ 5%). We determined these thresholds from our own dataset as well as frequency data from the 1000 Genomes Project European samples vOct2014 [43], Exome Aggregation Consortium non-Finnish European samples (ExAC v0.3 http://exac.broadinstitute.org) and the NHLBI GO Exome Sequencing Project European samples (http://evs.gs.washington.edu/EVS/). We used two complimentary methods to test for associations with variation in the *FKBP5* locus. First, we performed a SNP-set (Sequence) Kernel Association Test (SKAT) v1.0.9 [44], which employs a multivariate regression model that accommodates both risk and protective alleles in the tests of association. Second, we implemented a gene-burden test utilizing the previously described Combined Multivariate and Collapsing (CMC) method [45] to collapse all functional *FKBP5* variants by subject, and Firth’s penalized logistic regression via the logistf R package v1.21 [42] to test for association. Odds ratios were calculated from the covariate-corrected logistic regression output. The gene-level analyses examined either coding or regulatory variants. Broad coding variants included any disruptive variants and non-synonymous variants predicted to be damaging by any one of six software tools (SIFT [46], Polymorphism Phenotyping v2 (Polyphen-2) HDIV, Polyphen-2 HVAR [47], LRT [48], VEST [49] or MutationTaster [50]). Disruptive coding variants included any that are considered by ANNOVAR to be frameshift, stop-gain/nonsense or essential splice site mutations. Regulatory variants in the gene-level analyses were defined by their predicted ability to alter a regulatory region as determined by RegulomeDB v1.1 [51]. Specifically, broad regulatory variants had a RegulomeDB score of 1–6 and narrow regulatory variants had a more stringent RegulomeDB score of 1 or 2.

Sex and the first three principal components from the analysis of the GWAS data were included as covariates in all primary analyses. Statistical significance was calculated using permutation testing. We performed 10,000 permutations over the suicide attempter status using custom scripts.

### Genotyping

Replication of results were performed on bipolar disorder suicide attempters with definite/serious intent (N = 328) and bipolar disorder non-attempters (N = 655; S2 Table), which were obtained from the NIMH Genetics Initiative, (https://www.nimhgenetics.org/) [28] and did not overlap with the initial sample set.

Due to repetitive and homopolymer regions surrounding the top result, rs141713011, we used an RNase H-dependent (rhPCR) method (Integrated DNA Technologies, Coralville, IA, U.S.A.) to genotype this variant in the replication cohort. Briefly, we started with 20 ng genomic DNA and added SYBR GreenER qPCR SuperMix Universal (including ROX dye; Thermo Fisher Scientific, Waltham, MA, U.S.A.), specifically designed GEN2 primers for either the *A* allele or the *C* allele, and RNase H2 enzyme (Integrated DNA Technologies, Coralville, IA, U.S.A.). Real-time PCR was then performed on the ViiA7 system, and crossing threshold (Ct) values were used to identify sample genotypes.

The most commonly investigated *FKBP5* variant, rs3800373, was genotyped in the replication cohort using a Taqman SNP genotyping assay (C_27489960_10; Thermo Fisher Scientific, Waltham, MA, U.S.A.).

### Haplotype Analysis

To identify common haplotypes we utilized Haploview v4.2 [52]. Default values were employed except for the minimum MAF which was set at 0.05. Blocks were defined using “confidence intervals” [53].

## Results

### Dataset Quality

Following sequencing, 96.65% of the targeted sites in the *FKBP5* loci had at least ≥ 10X coverage and 100% of targets had at least ≥ 1X coverage (S1 Table). A total of 40,922 bases were covered by the designed probes and 565 variants were detected from this. The GATK quality control filters excluded 24 variants and our stringent quality control checks excluded a further 60 variants resulting in a final high quality dataset of 481 variants. This final dataset included 13 coding variants, 217 novel variants (not found in dbSNP 142 [54] or other sequencing databases) and 35 common variants (MAF > 0.05).

### Single-variant Analysis

**Fig 1:**
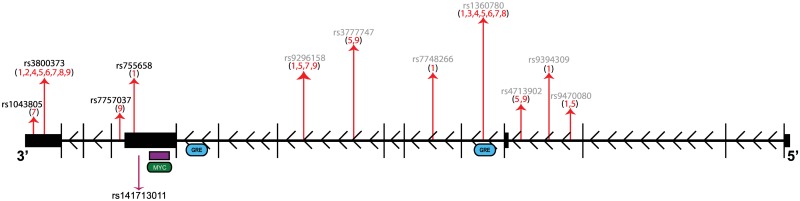
Schematic of the top variant in the *FKBP5* gene alongside previous findings. The top single variant result is displayed below the gene; variants or haplotypes with known associations to suicidal behavior are displayed above the gene. Gray variants were not covered by this study. Numbers represent references: **1.** [10] **2.** [14] **3.** [55] **4.** [12] **5.** [13] **6.** [11] **7.** [9] **8.** [56] **9.** [57]. All *FKBP5* transcripts have been collated to show exons (black vertical lines), introns (black horizontal lines) and untranslated regions (black boxes). A transcription factor binding site (TFBS; green box) and DNase hypersensitivity site (purple box) upstream of the top variant are labeled and are not to scale. GRE denotes glucocorticoid receptor response elements (blue boxes) and are not to scale.

Our primary analysis focused on 481 variants within or near *FKBP5* (S3 Table), and five of them were differentially represented among suicide attempters and non-attempters at a nominally significant level (Table 1). One variant, rs141713011, showed an excess of minor alleles in suicide attempters that was statistically significant following correction for multiple testing (Odds Ratio = 6.65, P-value = 7.5 x 10^−4^, Permuted P-value = 0.038). This variant resides in the intron or 3’UTR of multiple transcripts (Fig 1). The minor allele was present in sixteen suicide attempters (MAF = 0.017) and two non-attempters (MAF = 0.002).

**Table 1:** Single-variant results with a P-value < 0.05.

Variant^a^	Chromosomal Position^b^	Location^c^	P-value^d^	Permuted P-value	Odds Ratio^e^	Odds Ratio 95% Confidence Level		Minor Allele Frequency	
						Lower	Upper	Suicide Attempters	Non-Attempters
**rs141713011**	chr6:35553051	Intronic/3’UTR	7.5 x 10^−4^	0.038	6.65	2.056	33.62	0.017	0.002
**rs140664762**	chr6:35554071	Intronic/3’UTR	1.02 x 10^−3^	0.054	9.033	2.18	83.081	0.014	0.001
**rs575259136**	chr6:35691428	Intronic	0.015	0.60	0.078	0.001	0.67	0.00	0.006
**rs13192954**	chr6:35633456	Intronic	0.030	0.91	0.63	0.41	0.96	0.038	0.059
**rs553156199**	chr6:35543277	3'UTR	0.039	0.96	0.20	0.021	0.93	0.001	0.007

^a^Annotated from dbSNP 142.

^b^Using UCSC Genome Browser Human Feb. 2009 (GRCh37/hg19) Assembly.

^c^Including all transcripts as determined by UCSC Genome browser databases (UCSC Genes, GenCODE Genes, Ensembl Genes, RefSeq Genes and ENCODE databases).

^d^Corrected for sex and the first three principal components.

^e^Odds ratios shown are for the minor allele.

### Replication of Results

To further examine the top finding, rs141713011, we attempted to replicate the significant association with suicidal behavior in an additional cohort of 328 bipolar disorder suicide attempters and 655 bipolar disorder non-attempters. In this sample we observed five suicide attempters with the minor allele (MAF = 0.015) and ten non-attempters who carried it (MAF = 0.015). Thus, we failed to replicate our initial finding (Odds Ratio = 0.90, P-value = 0.78).

### Replication of Prior Findings

The two most implicated *FKBP5* variants in suicide are rs1360780 and rs3800373 (Fig 1). This study did not assess rs1360780, but it was covered in our previous attempted suicide GWAS and was not significantly associated with the phenotype there [16, 58].

In comparison, rs3800373 was assessed in our initial sample, and was not found to significantly differ between suicide attempters and non-attempters (MAF in suicide attempters = 0.28, MAF in non-attempters = 0.29, Odds Ratio = 0.94, P-value = 0.52, Permuted P-value = 1.00). Similarly, it was genotyped in the additional cohort, and again the results were not significant (MAF in suicide attempters = 0.30, MAF in non-attempters = 0.28, Odds Ratio = 1.07, P-value = 0.56).

### Sex-specific Analyses

To investigate if there was a sex-specific association between *FKBP5* and attempted suicide, we performed female-specific and male-specific single-variant analyses (S4 and S5 Tables). Three female-specific and four male-specific variants differed between suicide attempters and non-attempters at a nominally significant level (S6 Table). The previously discussed main finding (rs141713011) was also the top female-specific finding (Odds Ratio = 6.79, P-value = 8.1 x 10^−3^, Permuted P-value = 0.28). The top male-specific variant, rs13192954, showed an increase in significance compared to the main findings (Odds Ratio = 0.52, P-value = 0.026, Permuted P-value = 0.68). No sex-specific findings were statistically significant following correction for multiple testing.

### Gene-level Analysis

Due to the large amount of sequencing data produced from this study we were able to perform gene-level analyses to investigate whether *FKBP5* variants collectively influence suicidal behavior. To do this, we created four groups from the 481 variants studied based on their location and their ability to be damaging: **(i)** Regulatory Broad, **(ii)** Regulatory Narrow, **(iii)** Coding Broad and **(iv)** Coding Disruptive. The inclusion of two different MAF thresholds allowed us to investigate very rare and less rare separately.

We performed two distinct gene-level tests: SKAT and gene-burden. Both tests examine all *FKBP5* variants for each individual based on applied thresholds. The gene-burden test employs a logistic regression model to test the association between an indicator variable for whether individuals possess one or more rare, functional alleles of a specific category or not and suicide attempt. In comparison, SKAT employs a multivariate model that includes each variant as a covariate to look for significantly different distributions of the variants between suicide attempters and non-attempters, so it has the advantage of not being affected by the direction of effect on risk.

No gene-level tests showed evidence of association, regardless of minor allele threshold (Tables 2 and 3). The top result was from the coding broad analysis using the gene-burden approach (using either MAF threshold; P-value = 0.15). The top sex-specific P-value was identified using SKAT within the female Coding Broad subgroup (using either MAF threshold, P-value = 0.057).

**Table 2:** Gene-level results using two minor allele thresholds.

	SKAT P-value^a^		Burden P-value^a^		Odds Ratio	
	MAF 1%	MAF 5%	MAF 1%	MAF 5%	MAF 1%	MAF 5%
Regulatory Broad^b^	0.30	0.16	0.51	0.74	1.089	1.06
Regulatory Narrow^c^	0.29	0.32	0.80	1.00	1.047	1.00
Coding Broad^d^	0.37	0.37	0.15	0.15	0.41	0.41
Coding Disruptive^e^	N/A	N/A	0.45	0.45	0.32	0.32

N/A denotes values that could not be computed.

^a^Corrected for sex and the first three principal components.

^b^Predicted to be damaging to regulatory regions with a score of ≤ 6 by RegulomeDB.

^c^Predicted to be damaging to regulatory regions with a score of ≤ 2 by RegulomeDB.

^d^Predicted to be damaging to coding regions by at least one of six software programs (SIFT, Polyphen2 (HDIV and HVAR), LRT, MutationTaster and VEST) or considered disruptive.

^e^Considered to be an essential splicing variant, frameshift insertion/deletion or stop-gain variant using ANNOVAR.

**Table 3:** Sex-specific gene-level results using two minor allele thresholds.

	Female						Male					
	SKAT P-value^a^		Burden P-value^a^		Odds Ratio		SKAT P-value^a^		Burden P-value^a^		Odds Ratio	
	MAF 1%	MAF 5%	MAF 1%	MAF 5%	MAF 1%	MAF 5%	MAF 1%	MAF 5%	MAF 1%	MAF 5%	MAF 1%	MAF 5%
Regulatory Broad^b^	0.68	0.77	0.90	0.68	0.98	1.093	0.080	0.12	0.31	0.97	1.21	1.009
Regulatory Narrow^c^	0.23	0.71	0.91	0.83	0.97	0.96	0.67	0.52	0.70	0.77	1.10	1.057
Coding Broad^d^	0.057	0.057	0.10	0.10	0.25	0.25	0.74	0.74	0.71	0.71	0.73	0.73
Coding Disruptive^e^	N/A	N/A	0.46	0.46	0.32	0.32	N/A	N/A	N/A	N/A	N/A	N/A

N/A denotes values that could not be computed.

^a^Corrected for the first three principal components.

^b^Predicted to be damaging to regulatory regions with a score of ≤ 6 by RegulomeDB.

^c^Predicted to be damaging to regulatory regions with a score of ≤ 2 by RegulomeDB.

^d^Predicted to be damaging to coding regions by at least one of six software programs (SIFT, Polyphen2 (HDIV and HVAR), LRT, MutationTaster and VEST) or considered disruptive.

^e^Considered to be an essential splicing variant, frameshift insertion/deletion or stop-gain variant using ANNOVAR.

### Haplotype Analysis

**Fig 2:**
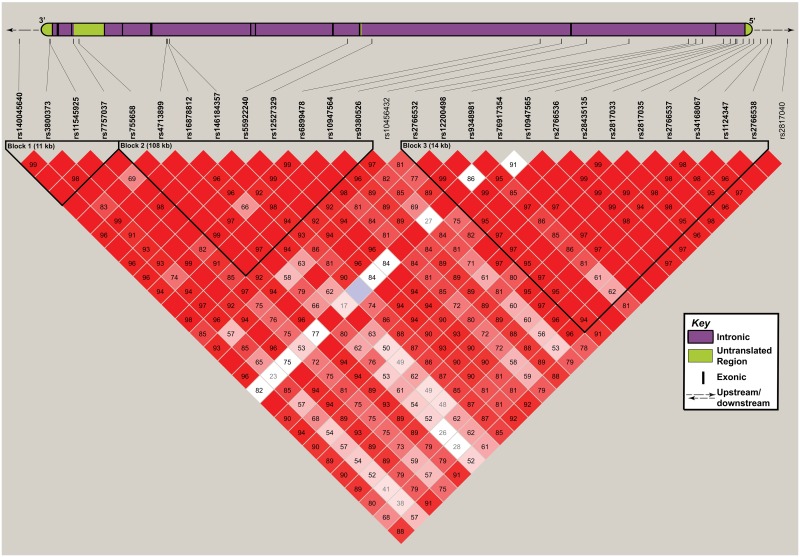
Haplotype block structures in the *FKBP5* region. A collated version of all *FKBP5* transcripts is represented at the top of the figure. Vertical black lines represent exons, green boxes represent untranslated regions and purple boxes represent intronic regions. The numbered squares display the D’ score, unnumbered squares have a D’ score of 1.0. Three haplotype blocks were generated and are enclosed by black lines. Lines connect the variants to their approximate location within the *FKBP5* locus.

Using Haploview we identified three haplotype blocks in the *FKBP5* region encompassing common variants (MAF > 0.05; Fig 2 and Table 4). Block 1 covered an 11 kb region encompassing four SNPs including the extensively studied rs3800373. Block 2 covered a 108 kb region and nine SNPs and Block 3 included thirteen SNPs over a 14 kb region. The top haplotype result was found within Block 3 (P-value = 0.097), but no significant associations between haplotypes and attempted suicide were identified.

**Table 4:** Haplotype results generated using Haploview.

	Haplotypes	Haplotype Frequency	Suicide Attempter Frequencies	Non-attempter Frequencies	Chi Square	Uncorrected P-value
**Block 1**	CCCA	0.45	0.46	0.45	0.71	0.40
	CCCC	0.26	0.26	0.27	0.15	0.70
	CACC	0.19	0.18	0.20	0.71	0.40
	AAAC	0.091	0.092	0.090	0.038	0.85
**Block 2**	CCCCCCCCC	0.52	0.51	0.52	0.089	0.77
	CACCCCACA	0.16	0.16	0.16	0.012	0.91
	CCCACCCAC	0.097	0.11	0.087	2.28	0.13
	ACACAAACA	0.092	0.093	0.091	0.015	0.90
	CCCCCCCAC	0.051	0.054	0.049	0.24	0.63
	CCCCCCACA	0.031	0.027	0.035	1.035	0.31
	CCACCCCCA	0.026	0.023	0.030	0.77	0.38
	CCCCCCCCA	0.021	0.016	0.026	2.45	0.12
**Block 3**	ACCCCACCACCCC	0.26	0.26	0.26	0.11	0.74
	CCACCCAACAACC	0.19	0.18	0.20	1.22	0.27
	CACCACCCCCCCC	0.18	0.18	0.18	0.00	1.00
	CCCCCCCACAAAA	0.17	0.18	0.17	0.46	0.50
	CCAACCAACAACC	0.062	0.070	0.055	1.69	0.19
	CCCCCCCCCCCCC	0.054	0.045	0.063	2.75	0.097
	CCACCCCACAAAA	0.024	0.025	0.022	0.19	0.66
	CCACCACCACCCC	0.014	0.017	0.011	1.37	0.24

All default values were used apart from the minimum minor allele frequency which was changed to 0.05.

## Discussion

Genetic variation can alter FKBP5 function, thereby affecting the co-chaperone complex essential to regulating translocation of the GR to the nucleus [8]. This may contribute to the over-activation of the HPA axis seen in subjects with suicidal behavior [5–7]. The goal of this study was to investigate whether variants within the *FKBP5* region, separately or together, influence suicidal behavior in individuals with bipolar disorder.

We employed the novel approach of sequencing both coding and regulatory regions using state-of-the-art next-generation sequencing technology. This allowed us to capture more functional areas of *FKBP5* and to focus on rare variation, thereby interrogating the gene more fully than our previous investigations could [16, 58]. Interestingly, the most significantly associated variant in this study was located near an area with experimentally demonstrated regulatory function (Fig 1) [35].

Our previous GWAS [16, 58] tested the hypothesis that common variants contributed to the risk for suicidal behavior, but no significant evidence to this effect was found in relation to *FKBP5*. This study, in contrast to our GWAS and most other *FKBP5* genetic studies, concentrated on both common and rare variation located in coding and regulatory regions of *FKBP5* and their contribution to the risk for attempted suicide. However, we were unable to find replicable associations with *FKBP5* variants individually or collectively.

As secondary analyses, we considered the role of sex-specific genetic variation in attempted suicide. There are several lines of evidence that suggest that suicidal behavior differs between females and males. Specifically, nearly twice as many males complete suicide compared to females [1] while more females attempt suicide [59, 60]. Furthermore, we have previously identified a female-specific common variant associated with suicidal behavior suggesting that sex-specific variants might influence the risk of this phenotype [16]. No statistically significant sex-specific results were found in this study. The female-specific SKAT coding broad test showed the top gene burden result (P = 0.057), but larger sample sizes are required to determine whether this trend is a true association.

Several candidate variants studies have previously implicated *FKBP5* variation in increasing risk for suicidal behavior. In a family study focused on bipolar disorder, four variants displayed increased significance when attempted suicide was included in the analysis as a covariate [9]. Another bipolar disorder study genotyped eight common *FKBP5* variants and found that a haplotype block containing seven of the variants was associated with attempted suicide [10]. A depression study focusing on two *FKBP5* variants found the *TT* genotype of rs1360780 and the *GG* genotype of rs3800373 were significantly associated with suicidal events, which included attempts, plans to attempt, and ideation, in adolescents on antidepressants; this finding remained even after controlling for treatment [11]. A similar study investigating selective serotonin reuptake inhibitor (SSRI) treatment genotyped rs1360780 and found that depressed individuals with the minor *T* allele were at increased risk of suicidal ideation following SSRI treatment [55]. Supporting evidence for these studies was provided by Menke et al. who investigated treatment-emergent suicidal ideation [27]. A recent study found three *FKBP5* variants were associated with suicide attempt, but not with suicide completion. They further identified a haplotype block with increased risk of suicidal behavior comparing depressed suicide attempters and depressed controls. However, these findings were not corrected for multiple testing [57]. A Japanese study identified a statistically significant haplotype block with two *FKBP5* variants (rs1360780 and rs3800373) in suicide completers, but did not find any single-variant associations [12]. A second, larger cohort of suicide completers was also genotyped for rs1360780 and rs3800373, and an association was found with rs3800373 [56]. It must be noted that the main limitations of all these studies was the small sample size and limited genotyping data. This was overcome in genome-wide studies which investigated suicidal behavior in larger samples and with broader coverage of the gene [16–27], but no significant associations were reported in *FKBP5* across these studies.

One variant of particular interest to the field, rs3800373 [9–14, 56, 57] has repeatedly been associated with suicidal behavior, but did not show association in our study (P-value = 0.52). This may be due to our case-only approach, which differs from the trio or case-control designs used by others. We utilized this approach to ensure that our results were not influenced by an underlying psychiatric disorder, but this did increase the level of suicidal ideation in the non-attempter cohort. Ideation has been previously associated with *FKBP5* variation [11, 55], and thus we may have reduced our power to detect an influence of *FKBP5*. Another possible explanation for the conflicting results may be that we focused on testing areas around and throughout the gene we surmised would be more likely to disrupt transcription or translation, and therefore we were unable to cover all the previously investigated variants (Fig 1). We did not cover intronic variants outside overlapping DNase hypersensitivity sites and transcription factor binding sites that have been associated with this phenotype. In particular, rs1360780, which can alter expression due to its proximity to a GR response element (GRE) [61], was not covered.

This study has other limitations. With an estimated 80% power (calculated using Quanto v1.2.4; http://biostats.usc.edu/Quanto.html), we were able to detect a single variant with an effect size as low as 2.35 assuming a study-wide significance of 1.04 x 10^−4^ and MAF of 0.05. For the replication analysis, we were able to detect a single variant with an effect size of 1.88 assuming a MAF of 0.05. With a MAF of 0.008 (seen in the replication results for rs141713011), an effect size of 3.70 can be detected. For the gene-level analyses, we had an estimated 80% power to detect an effect size as low as 1.95 assuming a study-wide significance of 6.3 x 10^−3^ and MAF of 0.05. In addition, it should be noted that other biological and environmental factors that impact suicidal behavior, such as epigenetics and early childhood trauma, were not included in this study, but should also be considered alongside these results. Considerable evidence has emerged for an epigenetic component to *FKBP5*’s role in suicidal behavior, with particular focus on demethylation within and around GREs [62]. Furthermore, interacting factors, such as high levels of early childhood trauma, have been shown to be positively associated with increased risk of suicide attempt [13, 14]. We did not have access to early childhood history for all subjects, so we could not investigate this gene-by-environment effect. An additional limitation of the replication dataset is the skewed proportion of male suicide attempters (15.85%; S2 Table) caused by the restricted number of remaining NIMH Genetics Initiative samples.

We have completed a large, targeted sequencing project focused on pursuing both coding regions and regulatory regions within *FKBP5*, a gene previously associated with suicidal behavior. While this study generated several nominally significant results and one statistically significant result, none of them survive correction for multiple testing and/or replication. Thus, it does not provide consistent support for a role of *FKBP5* variants increasing the risk of suicidal behavior. The failure to replicate the top finding reinforces the need for larger sample sizes in genetic studies. Greater datasets will more definitively test the relationship between *FKBP5* and suicidal behavior.

## Supporting Information

S1 Table Demographic characteristics of sample set. (DOCX)

S2 Table Demographic characteristics of the replication sample set. (DOCX)

S3 Table Single-variant results of all 481 *FKBP5* variants analyzed. (XLSX)

S4 Table Female-specific single-variant results of all 481 *FKBP5* variants analyzed. (XLSX)

S5 Table Male-specific single-variant results of all 481 *FKBP5* variants analyzed. (XLSX)

S6 Table Single-variant sex-specific results with a P-value < 0.05. (DOCX)

S7 Table Expanded dataset information for distribution and meta-analyses. (XLSX)

S1 File Additional institution information for sample collection. (DOCX)
