# Supplementary material for: Targeted Sequencing of FKBP5 in Suicide Attempters with Bipolar Disorder
Source: PLoS One. 2016 Dec 28;11(12):e0169158. doi: 10.1371/journal.pone.0169158 (PMC5193409; doi:10.1371/journal.pone.0169158)
Supplement: S2 Table — (DOCX) [file pone.0169158.s002.docx]

**S2 Table. Demographic characteristics of the replication sample set.**

|  | Suicide Attempters^a^ | Non-Attempters |
| --- | --- | --- |
| **Sex:**  ***Male/Female (Male%)*** | 52/276 (15.85%) | 301/354 (45.95%) |
| **Age:**  ***Average (Min-Max)*** | 41.65 (17-79) | 42.32 (17-83)^b^ |
| **Diagnosis:**  ***BP/SABP^c^*** | 271/57 | 607/48 |
| **Total number of subjects:** | 328 | 655 |

All subjects are unrelated and represent a homogenous ethnic population as determined by principal component analysis [1].

^a^With definite or serious intent.

^b^Two subjects did not provide age information.

^c^BPI denotes a bipolar disorder, type I diagnosis; SABP denotes a Schizoaffective Disorder, Bipolar Type diagnosis.

**Reference**

1. Willour VL, Seifuddin F, Mahon PB, Jancic D, Pirooznia M, Steele J, et al. A genome-wide association study of attempted suicide. Molecular psychiatry. 2012;17(4):433-44. doi: 10.1038/mp.2011.4. PubMed PMID: 21423239; PubMed Central PMCID: PMC4021719.
