# Supplementary material for: Targeted Sequencing of FKBP5 in Suicide Attempters with Bipolar Disorder
Source: PLoS One. 2016 Dec 28;11(12):e0169158. doi: 10.1371/journal.pone.0169158 (PMC5193409; doi:10.1371/journal.pone.0169158)
Supplement: S6 Table — (DOCX) [file pone.0169158.s006.docx]

**S6 Table. Single-variant sex-specific results with a P-value < 0.05.**

|  | Variant^a^ | Chromosomal Position^b^ | Location^c^ | P-value^d^ | Permuted  P-value | Odds Ratio^e^ | Odds Ratio 95% Confidence Level | | Minor Allele Frequency | |
| --- | --- | --- | --- | --- | --- | --- | --- | --- | --- | --- |
|  |  |  |  |  |  |  | **Lower** | **Upper** | **Suicide Attempters** | **Non-attempters** |
| Female | rs141713011 | chr6:35553051 | Intronic/3' UTR | 8.1 x 10^-3^ | 0.28 | 6.79 | 1.56 | 63.61 | 0.020 | 0.002 |
|  | rs147823312 | chr6:35688180 | 5’UTR | 0.012 | 0.40 | 0.074 | 0.001 | 0.63 | 0.00 | 0.012 |
|  | rs140664762 | chr6:35554071 | Intronic/3' UTR | 0.016 | 0.49 | 6.039 | 1.36 | 56.93 | 0.018 | 0.002 |
| Male | rs13192954 | chr6:35633456 | Intronic | 0.026 | 0.68 | 0.52 | 0.29 | 0.93 | 0.040 | 0.074 |
|  | rs575259136 | chr6:35691428 | Intronic | 0.027 | 0.70 | 0.090 | 0.001 | 0.80 | 0.00 | 0.011 |
|  | rs72913418 | chr6:35554243 | Intronic/3' UTR | 0.033 | 0.78 | 10.45 | 1.17 | 1376.38 | 0.011 | 0.00 |
|  | rs114600081 | chr6:35659025 | Intronic | 0.033 | 0.79 | 11.54 | 1.18 | 1547.48 | 0.009 | 0.00 |

^a^Annotated from dbSNP 142.

^b^Using UCSC Genome Browser Human Feb. 2009 (GRCh37/hg19) Assembly.

^c^Including all transcripts as determined by UCSC Genome browser databases (UCSC Genes, GenCODE Genes, Ensembl Genes, RefSeq Genes and ENCODE databases).

^d^Corrected for the first three principal components.

^e^Odds ratios shown are for the minor allele.
